# trans-Zeatin-N-glucosides have biological activity in *Arabidopsis thaliana*

**DOI:** 10.1371/journal.pone.0232762

**Published:** 2020-05-07

**Authors:** H. Tucker Hallmark, Martin Černý, Břetislav Brzobohatý, Aaron M. Rashotte

**Affiliations:** 1 Department of Biological Sciences, Auburn University, Auburn, AL, United States of America; 2 Department of Molecular Biology and Radiobiology, Faculty of AgriSciences, Mendel University in Brno, Brno, Czech Republic; Instituto de Biologia Molecular y Celular de Plantas, SPAIN

## Abstract

Cytokinin is an indispensable phytohormone responsible for physiological processes ranging from root development to leaf senescence. The term “cytokinin” refers to several dozen adenine-derived compounds occurring naturally in plants. Cytokinins (CKs) can be divided into various classes and forms; base forms are generally considered to be active while highly abundant cytokinin-N-glucosides (CKNGs), composed of a CK base irreversibly conjugated to a glucose molecule, are considered inactive. However, results from early CK studies suggest CKNGs do not always lack activity despite the perpetuation over several decades in the literature that they are inactive. Here we show that exogenous application of trans-Zeatin-N-glucosides (tZNGs, a specific class of CKNGs) to Arabidopsis results in CK response comparable to the application of an active CK base. These results are most apparent in senescence assays where both a CK base (tZ) and tZNGs (tZ7G, tZ9G) delay senescence in cotyledons. Further experiments involving root growth and shoot regeneration revealed tZNGs do not always have the same effects as tZ, and have largely distinct effects on the transcriptome and proteome. These data are in contrast to previous reports of CKNGs being inactive and raise questions about the function of these compounds as well as their mechanism of action.

## Introduction

Cytokinin (CK) is an adenine derivative which acts as a phytohormone and has roles in a variety of developmental processes, including shoot growth, senescence, and root growth [1]. However, the word “cytokinin” is an umbrella term which includes dozens of compounds naturally occurring in plants [2]. In *Arabidopsis thaliana* (hereafter referred to as Arabidopsis), abundant isoprenoid CKs fall into one of four classes: trans-Zeatin (tZ), isopentenyladenine (iP), cis-Zeatin (cZ), or dihydrozeatin (DZ) These four classes of CK are distinguished by their N6 side chains [2]. The side chains of tZ, cZ, and DZ are hydroxylated forms of the iP side chain; tZ and cZ differ from one another in their side chain stereochemistry, and DZ lacks a double bond in its side chain [2].

Within each class of CKs, several forms exist. For example, tZ exists in a ribophosphate form (tZRP), a riboside form (tZR), a base form (tZ), an O-glucoside form (tZOG), and N-glucoside forms (tZ7G, tZ9G) [2]. Each form of CKs has a different function: generally speaking, the ribophosphate form is a precursor to bioactive CKs [2], the riboside form is a transported form with some bioactivity [3,4], the base form is the canonical active form [3–5], the O-glucoside form serves as an inactive but convertible storage form [6–9], and N-glucosides have historically been referred to as being permanently inactive and irreversibly conjugated to a glucose molecule [2,7,10–12]. One exception to this is kinetin-N3-glucoside, which has been demonstrated to be reversibly conjugated [6]. Though these compounds are generally present in minuscule amounts, recent advances have allowed researchers to accurately measure CKs, and many studies in Arabidopsis have revealed that the majority of whole-plant CK is in the N-glucoside form [13–19].

Early work on cytokinin-N-glucosides (CKNG) revealed mixed results about the activity of these molecules; some bioassays performed using soybean suggested these compounds lacked activity, while other studies in *Raphanus* and *Amaranthus* suggested otherwise [20–22]. Perhaps the most insightful finding from these studies was that CKNGs have high metabolic stability and do not seem to be converted to bases, ribosides, or other CK forms typically considered to have biological activity [21–23].

In recent years, two genes from Arabidopsis, UGT76C2 and UGT76C1, have been identified as being glucosyltransferases responsible for conversion of CK bases to CKNGs [24]. Mutation of UGT76C2 leads to a significant reduction in CKNG, though they are not fully abolished, and plants harboring this mutation have increased sensitivity to the CK benzyladenine (BA), decreased seed size, and sensitivity to drought [25,26].

Recently, a metabolic study suggested that tZNGs are converted to active tZ in Arabidopsis within minutes of exogenous application [27] but it is important to note the fact that Hošek *et al*. used Arabidopsis cell lines for some of their work. CKNGs are largely localized to the extracellular space [28], so using a liquid-grown cell line may have significant impacts on CK homeostasis.

In this study, we examined the biological effects of trans-Zeatin-7-glucoside (tZ7G) and trans-Zeatin-9-glucoside (tZ9G), referred to collectively as tZNGs. We show that exogenous application of two highly abundant tZNGs can mimic exogenous application of a canonically active CK base, tZ, in senescence assays, where tZ7G and tZ9G delay senescence in detached leaves. However, these compounds do not appear to function as active CK in the inhibition of root growth or as a major factor in shoot regeneration like tZ. Interestingly, tZ7G and tZ9G appear to have many distinct transcriptional and proteomic regulatory targets separate from tZ. Since CKNGs have been previously shown to be non-convertible to CK base forms, this would indicate a different mechanism is responsible for the activity of tZ7G and tZ9G in CK bioassays. Together, these data suggest that tZNGs display context-dependent CK bioactivity, as well as having their own effects on gene expression.

## Materials and methods

### Plant material and growth conditions

Unless otherwise stated, all plants were wildtype Col-0 Arabidopsis. Seeds were surface sterilized using 70% ethanol and 20% bleach with Tween prior to plating on full strength MS agar plates. Seeds were stratified for 2–4 days before being moved into the growth chamber, which uses a 16h/8h light cycle (100μE) with temperatures of 22°C/18°C. Mutant *cypDM* seed was provided by RIKEN BRC through the National BioResource Project of the MEXT/AMED, Japan.

### Cotyledon senescence assay

The cotyledon senescence assays were modified for use with Arabidopsis from previously established CK bioassays [29,30] and were performed at least three times. Cotyledons (15 per treatment) of wildtype Col-0 Arabidopsis seedlings were excised 12 day after germination at the petiole and floated on 3mM MES buffer at pH 5.7. Cytokinin (tZ, tZ7G, tZ9G) was added to a final concentration of 1μM (with the exception of in the dose curve experiments in which the indicated concentration was used) or an equivalent volume of DMSO was added as a solvent control. CKNGs used in this study were obtained from OlChemIm (Olomouc, Czech Republic) as analytical standards with >95% purity guaranteed; no CK bases contaminated these standards. Previous work with CKNGs obtained from OlChemIm has revealed there is no conversion to active CKs during chlorophyll retention assays [31]. Once in solution, the cotyledons were placed in the dark at 20°C for six days, at which point chlorophyll was extracted and quantified according to a previously established protocol [32]. Briefly, cotyledons were transferred to twenty volumes of methanol and were placed at 4°C overnight. The next morning, 100μL of each solution was added to 900μL methanol and absorbance was measured at A652 and A665 to quantify chlorophyll concentration.

### Root growth inhibition assay

Wildtype Col-0 Arabidopsis seedlings were germinated on full strength MS agar. Four days after germination, seedlings of uniform size were transferred to full strength MS plates supplemented with 1μM cytokinin (tZ, tZ7G, tZ9G) or an equivalent volume (0.1%) DMSO. The transferred seedlings were allowed to continue to grow under standard conditions until day 9. Root growth from day 4 to day 9 was measured using ImageJ (NIH). Three biological replicates consisting of at least ten plants per treatment were performed.

### Shoot regeneration assay

Shoot regeneration assay was completed similarly to previous assays [33]. Wildtype Col-0 Arabidopsis seedlings were grown for five days in the dark for the seedlings to become etiolated. The hypocotyl was excised, avoiding tissue within 2mm of the root-shoot junction and cotyledons. Hypocotyls were then placed on full strength MS agar supplemented with 5μM cytokinin (tZ, tZ7G, tZ9G) or an equivalent volume of DMSO (0.5%) and 5μM NAA. Development was tracked over 45 days and final callus masses were measured. Three biological replicates consisting of at least four calli per treatment were performed.

### Transcriptomic analysis

Whole Col-0 Arabidopsis seedlings, ten days after germination, were transferred from MS agar plates to petri dishes containing 3mM MES buffer at pH 5.7 supplemented with either 1μM cytokinin (tZ, tZ7G, tZ9G) or an equivalent volume of DMSO (0.1%). Plant were gently shaken during a two-hour treatment before seedlings were removed and snap-frozen in liquid nitrogen. RNA was extracted using a Qiagen RNeasy Plant Mini Kit, according to manufacturer’s instructions. RNA was sent to Novogene, Inc. for quality check, library preparation, and 150x paired end sequencing on an Illumina HiSeq X. At least 20 million paired end reads were generated per sample. Each treatment had three biological replicates with at least 5 seedlings per treatment. Read quality was evaluated with FastQC [34]. Trimming was performed using Trimmomatic [35]. Read mapping, transcript assembly, and differential expression analysis was performed using HISAT2, StringTie, and DESeq2 [36,37]. The significance threshold was padj < 0.05. GO analysis was performed using AgriGO 2.0 [38]. Raw sequence data is available for download at NCBI Sequence Read Archive under the BioProject ID PRJNA588257. qRT-PCR confirmation of RNAseq data was completed using SYBR-green and sequence-specific primers on an Eppendorf Realplex2 as previously described [39]. Subcellular localization analysis was performed by running DEG lists through SUBA4 and using the SUBA consensus location [40]. Full transcriptome results and primer sequences can be found in Supplementary Information.

### Protein extraction and LC-MS analysis

Plants for proteomic analysis were grown and treated in a manner similar to that done for transcriptomic analysis, then flash frozen in liquid analysis for further sample preparation as detailed above. Each treatment had four biological replicates. Total protein extracts were prepared as described previously [41] with some modifications. Briefly, 100 mg of homogenized tissue was precipitated with 1 ml of methanol/methyl tert-butyl ether/water (1:3:1), pellets were solubilized [2% (w/v) SDS, 30% (w/v) sucrose, 5% (v/v) β-mercaptoethanol, 5 mM EDTA, 100 mM Tris, pH 8.0], proteins were extracted with phenol, precipitated, and the resulting pellets were solubilized. Next, aliquots corresponding to 100 μg of protein were reduced, alkylated with iodoacetamide, digested with trypsin (1:100, Promega) and desalted by C18 SPE. Finally, aliquots corresponding to 2.5 μg of peptide were analyzed by nanoflow C18 reverse-phase liquid chromatography using a 15 cm column (Zorbax, Agilent), a Dionex Ultimate 3000 RSLC nano-UPLC system (Thermo) and the Orbitrap Fusion Lumos Tribrid Mass Spectrometer. Peptides were eluted with up to a 120-min, 4% to 40% acetonitrile gradient. Spectra were acquired using the default settings for peptide identification, employing HCD activation, resolution 60000 (MS) and 15000 (MS2), and 60 s dynamic exclusion. The measured spectra were recalibrated and searched against Araport 11 protein database by Proteome Discoverer 2.3, employing Sequest HT, Mascot 2.4 and MS Amanda 2.0 with the following parameters: Enzyme—trypsin, max two missed cleavage sites; MS1 tolerance—5 ppm; MS2 tolerance—15 ppm (MS Amanda), 0.1 Da (Sequest, Mascot); Modifications—carbamidomethyl (Cys) and up to three dynamic modifications including Met oxidation, Asn/Gln deamidation, N-terminal acetylation. Only proteins with at least two unique peptides were considered for the quantitative analysis. The quantitative differences were determined by Minora, employing precursor ion quantification followed by normalization and background based t-test, and the resulting data were evaluated in Skyline 19.1 (MacCossLab Software, https://skyline.gs.washington.edu).The mass spectrometry proteomics data have been deposited to the ProteomeXchange Consortium (http://proteomecentral.proteomexchange.org) via the PRIDE partner repository (Vizcanzio et al., 2016) with the dataset identifier PXD016087.

## Results

### trans-Zeatin-N-glucosides delay senescence in detached cotyledons

**Fig 1:**
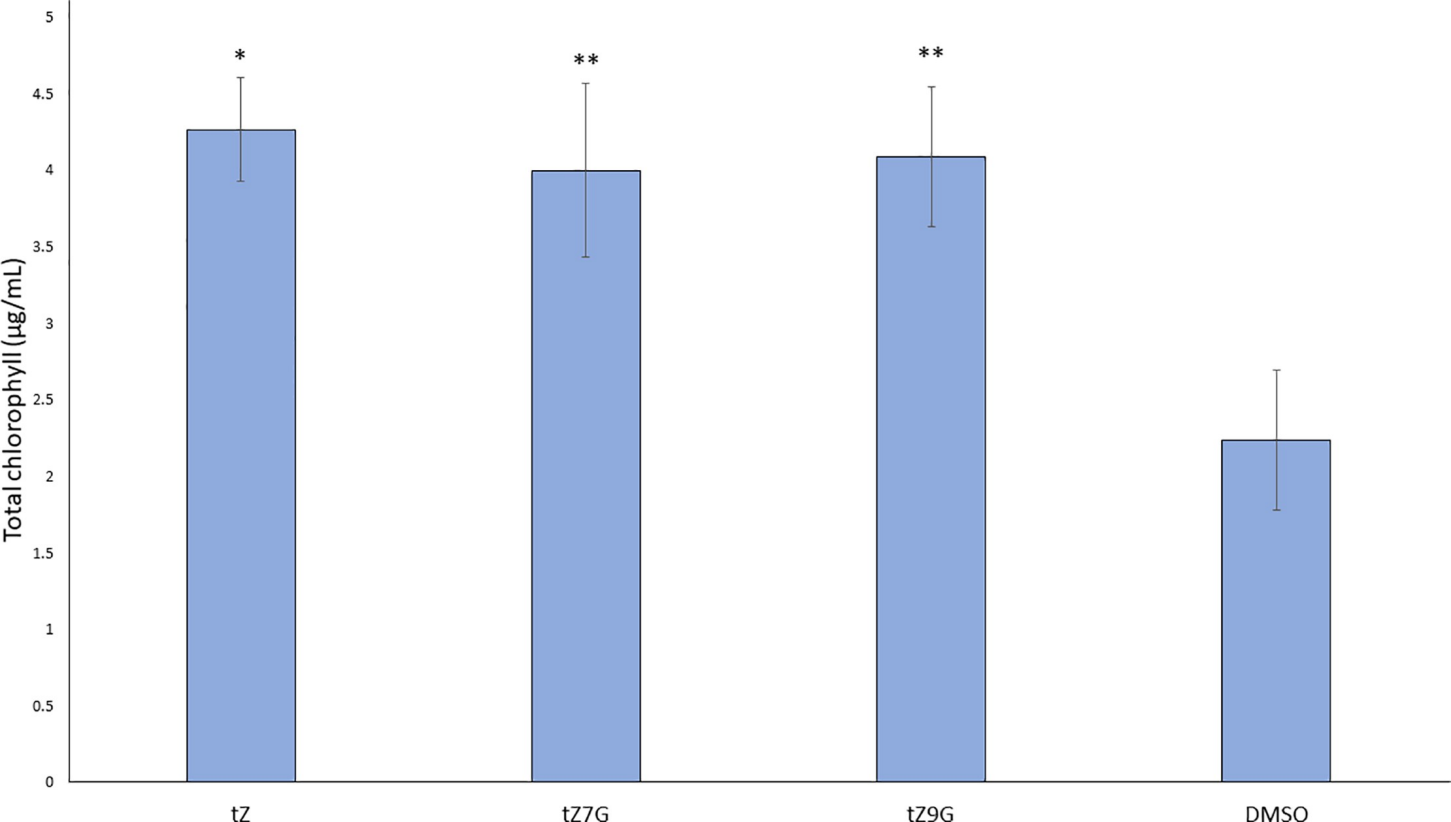
trans-Zeatin-N-glucosides delay chlorophyll degradation in detached cotyledons. Cotyledons from 12dag WT Arabidopsis seedlings were excised and floated abaxial side down in 3mM MES buffer (pH 5.7) supplemented with 1μM of the indicated hormone or 0.1% DMSO as a control. Solutions were placed in the dark for six days, after which chlorophyll was extracted and quantified according to Sumanta et al., 2014. Average ± SE of three biological replicates is presented. * p-value < 0.05, ** p-value < 0.01, paired two-tailed Student’s t test.

**Fig 2:**
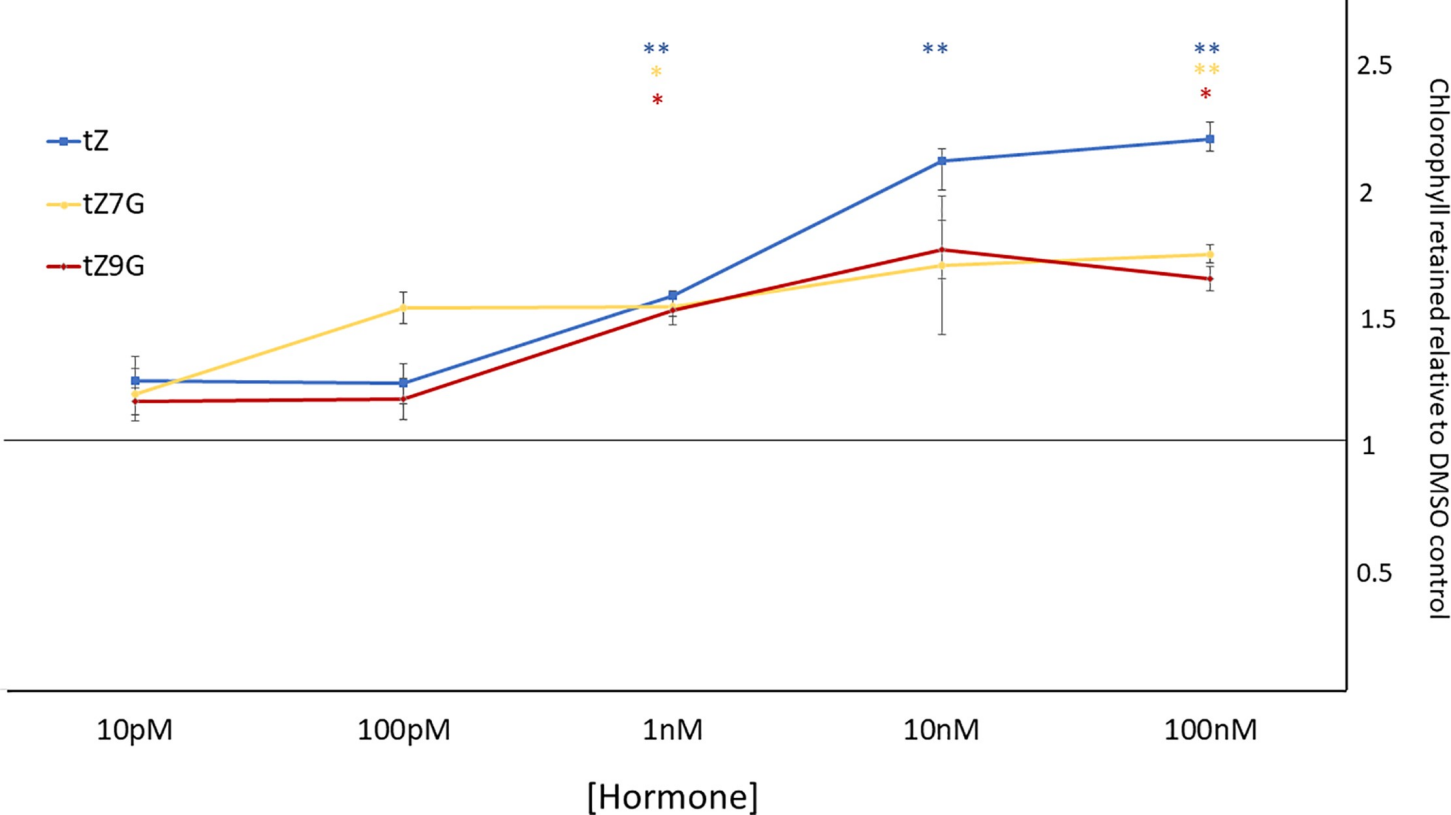
trans-Zeatin-N-glucosides delay chlorophyll degradation in detached cotyledons in a dose-dependent manner. Experiment performed identically to that in Fig 1 but with different concentrations of hormones. Average ± SE of three biological replicates is presented as a normalized level of chlorophyll relative to the chlorophyll content in the DMSO control cotyledons. Significance was determined by a two-way ANOVA (treatment factor, p-value = 0.0005) followed by post-hoc t-tests comparing hormone treatments to DMSO control. * p-value < 0.05, ** p-value < 0.01. Significance asterisk colors correspond to the line of the same color.

Active cytokinins are known to delay senescence in detached cotyledons [21,29,42,43]. Chlorophyll content can be a proxy to determine if a leaf has senesced or if senescence has been delayed because chlorophyll degradation occurs during senescence [44]. In this assay, cotyledons from 12 day after germination (12dag) seedlings were floated in buffer supplemented with 1μM tZ, tZ7G, or tZ9G. Hormones were dissolved in DMSO (0.1% DMSO treatment was included as a solvent control). The treated cotyledons were placed in the dark for six days to allow senescence to take place. As expected, cotyledons treated with the known active cytokinin tZ retained significantly more chlorophyll than the solvent control (Fig 1). Interestingly, both tZ7G- and tZ9G-treated samples also retained similar levels of chlorophyll (Fig 1). To test if this effect was dose dependent, a range of hormone concentrations were tested, starting at 10pM (Fig 2). Both tZ7G and tZ9G retained more chlorophyll than the DMSO control even at 1nM concentration (Fig 2). This indicates that tZ7G and tZ9G are effective in this bioassay at similar levels as tZ.

### trans-Zeatin-N-glucosides do not affect root elongation

**Fig 3:**
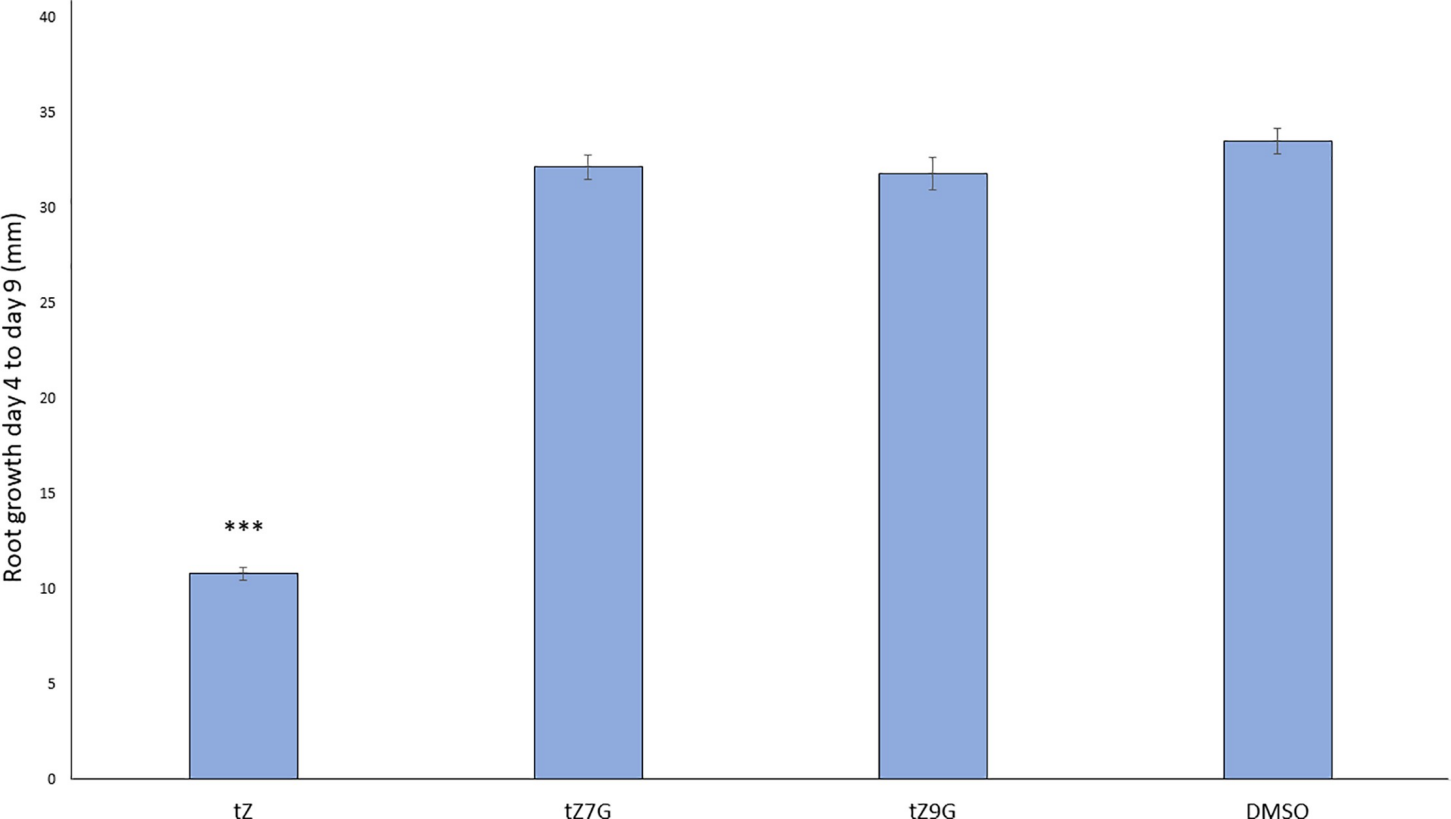
trans-Zeatin-N-glucosides do not inhibit root growth. Arabidopsis seedlings were transferred from standard MS + 1% sucrose at 4dag to the same media supplemented with 1μM of the indicated hormone or 0.1% DMSO as a control. Root growth since being transferred to the new media was measured on day 9. Average ± SE of three biological replicates is presented (n > 10 per treatment per replicate). *** p-value < 0.001, Student’s two-tailed t test.

Active cytokinins are known to inhibit root elongation [45,46]. Here, seedlings were germinated on standard media and transferred to the same media supplemented with 1μM tZ, tZ7G, tZ9G, or a 0.1% DMSO solvent control. Root growth was measured from day 4, when the seedlings were transferred to the new media, until day 9. The tZ-treated seedlings had significantly inhibited root growth relative to the DMSO control, whereas the tZNG-treated seedlings saw no difference from the control at the tested concentration (Fig 3). This suggests tZ7G and tZ9G do not significantly influence root elongation in seedlings of this stage.

### trans-Zeatin-7-glucoside modestly influences shoot regeneration

**Fig 4:**
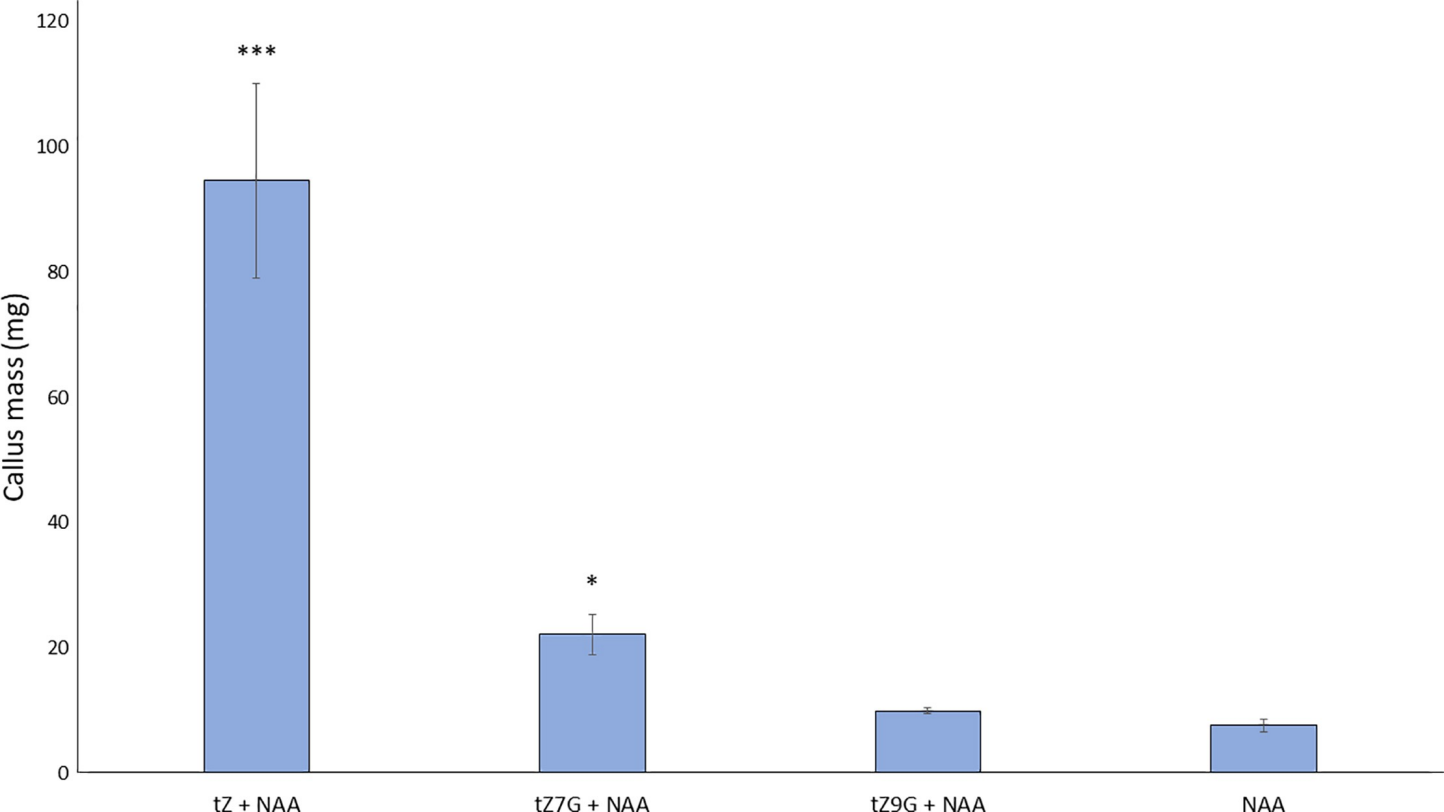
Trans-Zeatin-7-glucoside mildly promotes shoot initiation. Hypocotyls from etiolated Arabidopsis seedlings were transferred to MS + 1% sucrose supplemented with equimolar amounts of NAA and the indicated cytokinins. The negative controls were supplemented with an equal volume of DMSO in lieu of cytokinin. After 45 days, callus weight was measured. Average ± SE of three biological replicates is shown (n ≥ 4 per treatment per replicate). *** p-value < 0.001, * p-value < 0.05, Student’s two-tailed t test.

A classical test of cytokinin activity is the shoot regeneration assay in which hypocotyls are exposed to cytokinin and auxin. If the cytokinin is “active” and present in proper concentration, then a callus should develop, begin to turn green, and produce shoot-like structures such as leaves [47]. In this assay, hypocotyls were excised from the etiolated Arabidopsis seedlings and were subjected to a treatment with the synthetic auxin NAA and an equal amount of tZ, tZ7G, tZ9G, or DMSO as a negative control. Hormone concentrations used in this experiment were chosen based on Rashotte et al., 2006. To quantify shoot regeneration, callus weight was determined after 45 days on the hormone-containing media. Samples treated with NAA and tZ produced significantly higher callus weight than those with NAA and DMSO (Fig 4). Though the difference is modest, samples treated with NAA and tZ7G also had a slight but statistically significant increase in weight, relative to the control treatment. No significant change was seen in tZ9G samples.

### trans-Zeatin-N-glucosides alter the transcriptome distinctly from trans-Zeatin

**Fig 5:**
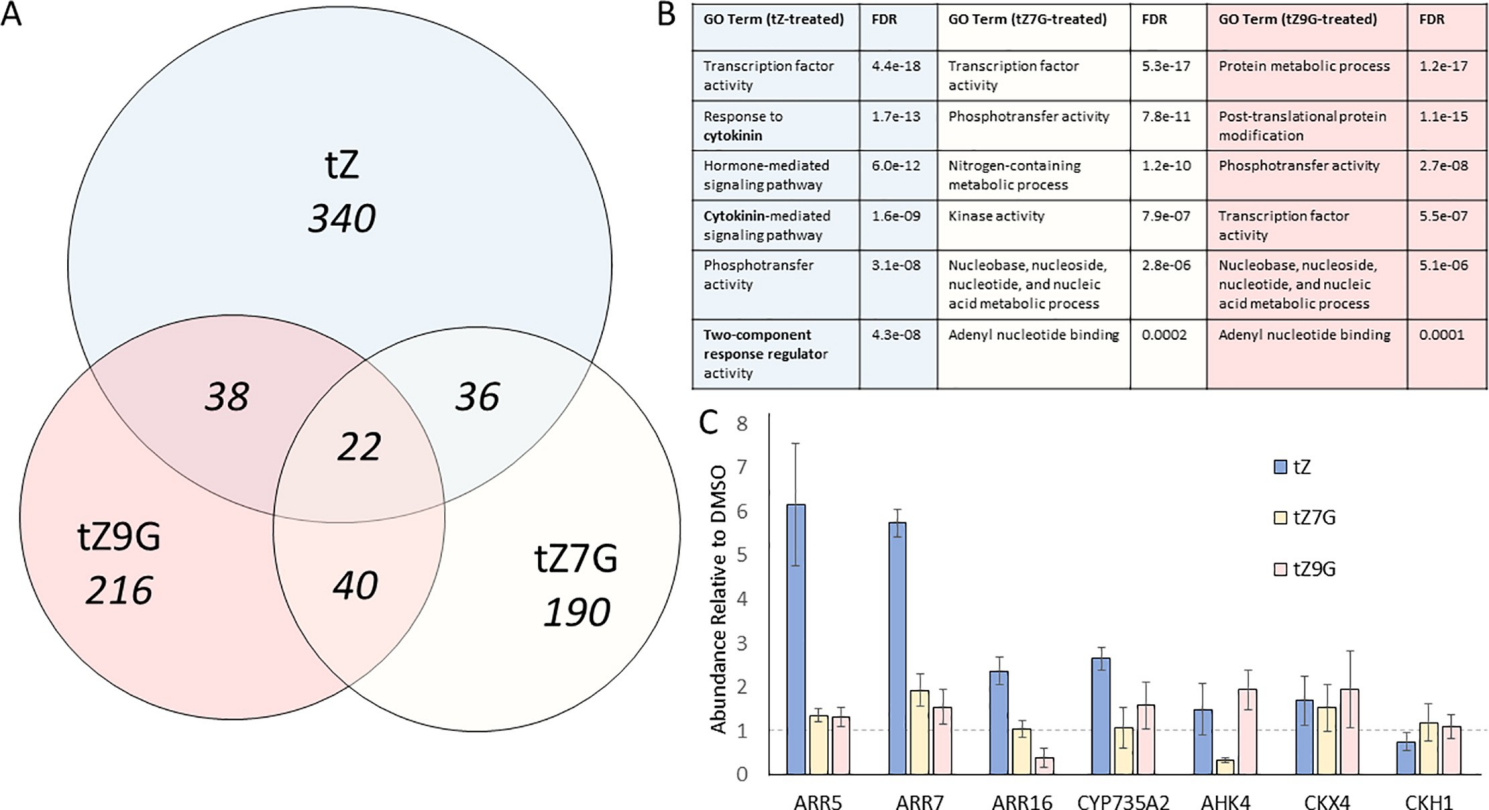
trans-Zeatin-N-glucosides alter the Arabidopsis transcriptome distinctly from trans-Zeatin. A, Venn diagram showing number of differentially expressed transcripts after two-hour treatment of 10dag WT Arabidopsis seedlings with 1μM hormone. B, tZ-treated seedlings are enriched for CK-related Gene Ontology terms, unlike tZ7G- and tZ9G-treated seedlings. C, qRT-PCR confirmation of RNAseq results; data presented is average ± SE of three independent biological replicates. Treatment with tZ7G or tZ9G does not mimic treatment with tZ with regards to regulation of most tested CK-related genes.

To determine if tZNGs affect the transcriptome of Arabidopsis, RNA sequencing was performed. Ten-day old seedlings were floated in buffer supplemented with 1μM tZ, tZ7G, tZ9G, or a 0.1% DMSO control treatment for two hours. After the two-hour treatment, tissue was flash frozen in liquid nitrogen and RNA was extracted and sequenced. The sequencing data was mapped to the Arabidopsis reference genome, and transcript level differential expression analysis was performed to determine Differentially Expressed Gene (DEG) lists for comparison. Each treatment group had hundreds of differentially expressed transcripts, but overlap between treatments was minimal (Fig 5A). Treatment with tZ, tZ7G, and tZ9G led to 340, 190, and 216 transcripts being uniquely regulated, respectively (Fig 5A). The regulation of cytokinin-related DEGs are presented in Table 1, revealing tZ to induce most of these. To validate the overall findings of the RNAseq analysis, qRT-PCR was performed and results generally mirrored transcriptome findings (Fig 5C). These results suggest tZNGs do not tend to regulate CK-related genes in a similar manner to tZ, though there are some notable exceptions such as CKX4 (Table 1, Fig 5C).

**Table 1:** List of select differentially expressed CK-related transcripts after two-hour treatment with tZ, tZ7G, and tZ9G relative to the DMSO control. Values in bold indicate a significant difference (adjusted p-value < 0.05) between the CK-treated and control-treated samples. NA indicates read counts that were too low to establish statistical significance.

Gene Identifier	Gene Name	tZ		tZ7G		tZ9G		CK Function
		log2FC	padj	log2FC	padj	log2FC	padj	
AT2G01830.4	AHK4	**1.31**	**1.05E-07**	0.77	0.99	0.53	1	Receptor
AT2G01830.6	AHK4	20.78	NA	1.87	NA	**9.75**	**9.27E-11**	Receptor
AT1G59940.1	ARR3	**6.39**	**0.004**	1.47	NA	3.76	1	Response Regulator
AT1G10470.2	ARR4	**2.37**	**2.74E-33**	0.09	1	0.29	1	Response Regulator
AT3G48100.1	ARR5	**3.17**	**5.60E-14**	0.17	1	0.15	1	Response Regulator
AT5G62920.1	ARR6	**3.01**	**1.31E-17**	0.64	1	0.37	1	Response Regulator
AT1G19050.1	ARR7	**2.8**	**3.79E-14**	-0.21	1	0.01	1	Response Regulator
AT2G41310.1	ARR8	**2.1**	**2.46E-05**	0.2	1	0.4	1	Response Regulator
AT1G74890.1	ARR15	**4.05**	**1.34E-09**	-0.29	1	0.01	1	Response Regulator
AT2G40670.1	ARR16	**1.52**	**1.83E-06**	-0.6	1	-0.13	1	Response Regulator
AT3G44326.1	CFB	**2.07**	**0.001**	-0.04	1	0.27	1	Signaling F-Box
AT2G46310.1	CRF5	**1.56**	**0.002**	-0.13	1	-0.02	1	Signaling CRF
AT1G17440.1	CKH1	1.05	0.366	**1.75**	**0.002**	**1.53**	**0.048**	Signaling Response
AT2G17820.1	AHK1	**1.36**	**6.17E-13**	-0.1	1	0.01	1	Ortholog of Receptor
AT5G05860.1	UGT76C2	**1.56**	**3.34E-07**	-0.27	1	-0.01	1	N-Conjugation
AT1G67110.1	CYP735A2	**3.01**	**3.96E-13**	0.19	1	0	1	Biosynthesis
AT5G56970.1	CKX3	**3.13**	**0.029**	0.52	1	0	1	Degradation
AT4G29740.3	CKX4	**6.59**	**5.94E-16**	**4.16**	**0.027**	3.01	0.907	Degradation

To better understand the generated gene lists, Gene Ontology (GO) term enrichment was performed using agriGO v2.0 [38]. As expected, the tZ-treated group had several GO terms associated with cytokinin (Fig 5B). The tZ7G and tZ9G groups did not have any directly cytokinin-related GO terms, but did include “Nucleobase, nucleoside, nucleotide, and nucleic acid metabolic process,” and “Adenyl nucleotide binding” (Fig 5B). It is worth noting that tZ, tZ7G, and tZ9G are all adenine-derived compounds. Although DEGs from neither tZ7G nor tZ9G were enriched for CK-related GO terms, it is of particular interest that tZ9G induced the CK receptor AHK4, specifically a transcript isoform with an extended 5’UTR, compared to the isoform induced by tZ (Table 1). Both tZNGs induced CKH1, a histidine kinase that regulates CK response [48,49], and tZ7G induced CKX4, a cytokinin degrading enzyme which has been shown to degrade CK bases and ribosides, but likely not CKNGs [50,51] (Table 1). Several other potentially interesting transcripts were found as DEGs after tZ7G and tZ9G treatments (S2 Table). These include SEN2/AtCAT3, a catalase gene connected to senescence response, which was repressed in tZ7G and tZ9G as well as in tZ; PAC (Pale Cress), a gene involved in chloroplast and leaf development, which was repressed in tZ7G and tZ9G only; and SOD1 (superoxide dismutase 1) that was induced only in tZ9G treatment (S2 Table) [52–54]. While subcellular localization of DEGs appears to be quite similar between all CK treatments, there is a slight increase in plastid localization for tZNG treatment, relative to tZ treatment (S1 Fig).

### trans-Zeatin-N-glucosides alter the proteome distinctly from trans-Zeatin

**Fig 6:**
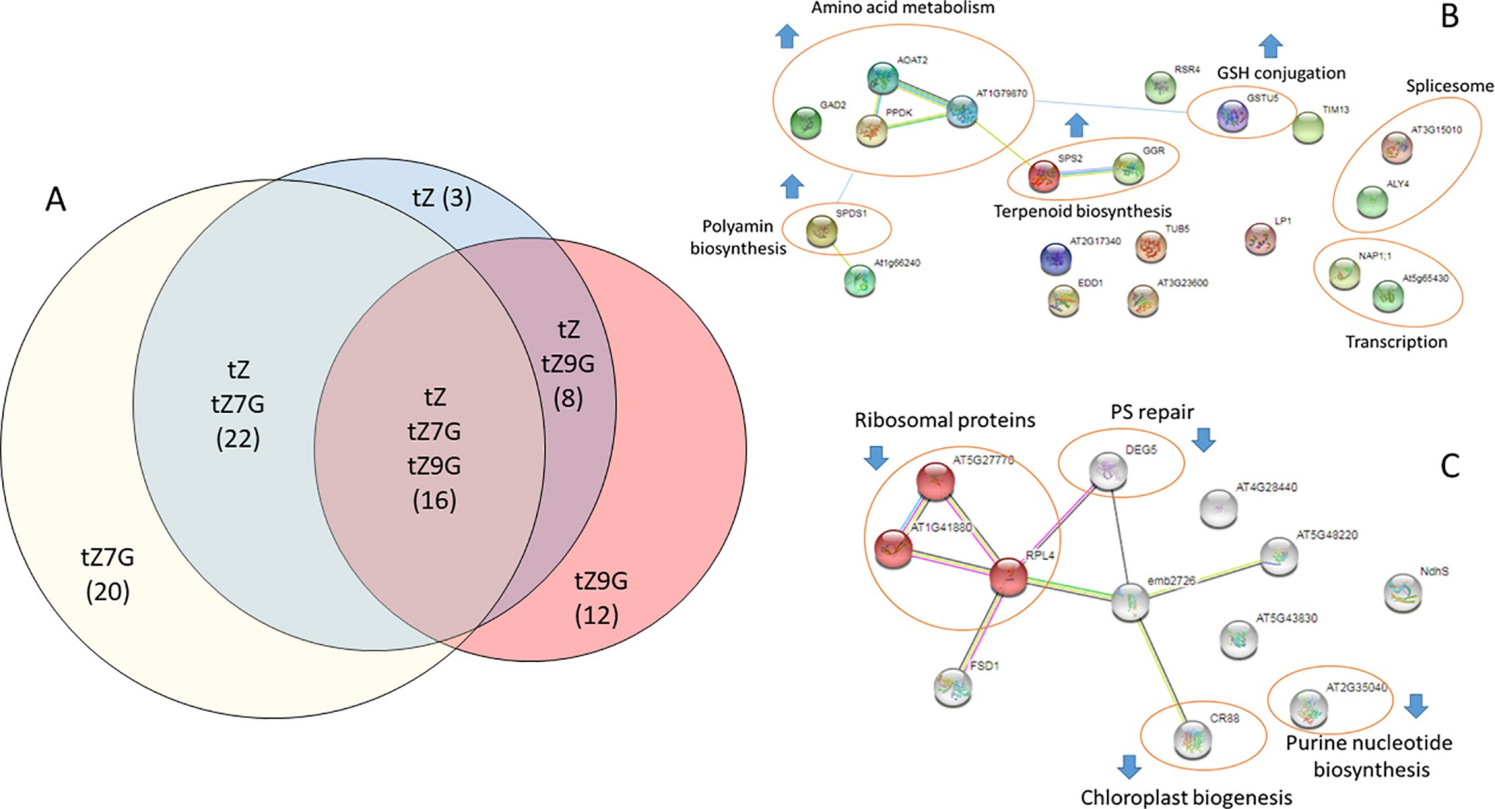
trans-Zeatin-N-glucosides alter the Arabidopsis proteome in a partially overlapping manner with trans-Zeatin. A, Proportional Venn diagram showing number (in parentheses) of differentially expressed proteins after two-hour treatment of 10dag WT Arabidopsis seedlings with 1μM hormone. B, Treatment with tZ7G affects amino acid metabolism, terpenoid biosynthesis, and other pathways. C, Treatment with tZ9G affects largely plastid proteins, including those involved in chloroplast biogenesis and photosystem (PS) repair.

In order to determine if tZNGs might be connected to gene regulation beyond the transcriptional level, a proteomic analysis was performed. Seedlings were treated with tZ, tZ7G, tZ9G, or a DMSO control in the same manner as for the transcriptome analysis, then frozen in liquid nitrogen for additional preparation and examination. Once prepared, 2119 protein families were identified, and the relative abundances of 14,412 peptides representing 1,629 proteins were quantified. The liquid chromatography/mass spectrometry (LC/MS) shotgun proteomic analysis yielded 81 proteins with a significant (P < 0.05; background based t-test) difference from DMSO-treated control samples in three biological replicates. In detail, 49, 58, and 36 proteins were identified to be regulated by tZ, tZ7G, and tZ9G, respectively (Fig 6). There was a nearly even split between induced (42) and repressed (39) proteins (S1 Table). The list of tZ-regulated proteins shares some overlap with previously noted CK-regulated proteins from other proteomic analyses (Table 2) [55–60]. While there is only moderate overlap between the significantly regulated proteins between tZ7G and tZ9G (only 16 shared between the two treatments), both treatments overlap heavily with tZ (Fig 6). Each treatment had several uniquely regulated proteins.

**Table 2:** List of select differentially expressed proteins after two-hour treatment with tZ, tZ7G, and tZ9G relative to the DMSO control. Numbers in bold and italicized indicate a statistically significant difference in the abundance of the protein between the CK treatment and the DMSO control, background based t-test p-value < 0.05.

Accession	Response	Protein name (UniProt)	Relative abundance tZ vs DMSO	Relative abundance tZ7G vs DMSO	Relative abundance tZ9G vs DMSO	Gene Symbol	# Unique Peptides	Previous studies showing CK-regulation	UniProt Accession
**AT3G26450**	**tZ- | tZ7G- | tZ9G-**	***Polyketide cyclase/dehydrase and lipid transport superfamily protein***	***0*.*3***	***0*.*5***	***0*.*5***	**AT3G26450**	**6**	**Lochmanová et al., 2008; Černý et al., 2013; down**	**Q9LIN0**
**AT4G10300**	**tZ- | tZ7G- | tZ9G-**	***RmlC-like cupins superfamily protein***	***0*.*2***	***0*.*2***	***0*.*4***	**AT4G10300**	**2**	**Černý et al., 2014; down**	**Q9SV91**
**AT4G25050**	**tZ- | tZ7G- | tZ9G-**	**Acyl carrier protein 4, chloroplastic**	***0*.*5***	***0*.*6***	***0*.*3***	**ACP4**	**3**	**Černý et al., 2013; up**	**Q9SW21**
**AT1G76180**	**tZ- | tZ7G- | tZ9G-**	**Dehydrin ERD14**	***0*.*3***	***0*.*2***	***0*.*2***	**ERD14**	**5**	**Černý et al., 2011; Černý et al., 2014; down / up**	**P42763**
**AT3G14290**	**tZ+ | tZ7G+ | tZ9G+**	**Proteasome subunit alpha type-5-B, EC 3.4.25.1 (20S proteasome alpha subunit E-2) (Proteasome component Z)**	***2*.*2***	***2*.*2***	***1*.*4***	**PAE2**	**3**	**Černý et al., 2011; down**	**Q42134**
**AT2G45180**	**tZ+**	***At2g45180 (At2g45180/T14P1*.*1) (Bifunctional inhibitor/lipid-transfer protein/seed storage 2S albumin superfamily protein) (Expressed protein) (Putative proline-rich protein)***	***2*.*9***	**1.6**	**0.8**	**AT2G45180**	**2**	** **	**Q42044**
**AT3G15450**	**tZ-**	***Aluminum induced protein with YGL and LRDR motifs***	***0*.*1***	**0.5**	**0.3**	**AT3G15450**	**2**	** **	**F4IYS4**
**AT2G24940**	**tZ-**	**Probable steroid-binding protein 3, AtMP3 (Membrane-associated progesterone-binding protein 2, AtMAPR2)**	***0*.*2***	**0.6**	**0.7**	**MAPR2**	**2**	** **	**Q9SK39**
**AT3G48110**	**tZ7G+**	**Glycine—tRNA ligase, chloroplastic/mitochondrial 2, EC 6.1.1.14 (Glycyl-tRNA synthetase 2, GlyRS-2) (Protein EMBRYO-DEFECTIVE-DEVELOPMENT 1)**	**2.8**	***4*.*5***	**0.7**	**EDD1**	**2**	**Žďárská et al., 2013; up**	**Q8L785**
**AT4G26110**	**tZ7G+**	**Nucleosome assembly protein 1;1, AtNAP1;1 (Nucleosome/chromatin assembly factor group A1)**	**1.1**	***0*.*5***	**0.8**	**NAP1;1**	**4**	**Černý et al., 2011; Zhang et al., 2012; up**	**Q9SZI2**
**AT1G20010**	**tZ7G-**	**Tubulin beta-5 chain (Beta-5-tubulin)**	**0.7**	***0*.*3***	**0.7**	**TUB5**	**5**	**Černý et al., 2011; up**	**P29513**
**AT5G43830**	**tZ9G-**	***Aluminum induced protein with YGL and LRDR motifs***	**0.9**	**1.1**	***0*.*4***	**AT5G43830**	**5**	**Černý et al., 2011; Černý et al., 2014; down**	**Q9FG81**

Interestingly, tZ, tZ7G, and tZ9G treatment all led to the differential regulation of ACP4, ERD14, PAE2, AT3G26450, AT4G10300, which have all previously been shown to be CK responsive proteins [55–58] (Table 2). The three proteins regulated by tZ, but not tZNGs, were MAPR2, AT3G15450, AT2G45180, none of which appear to be functionally characterized to date. Among the protein regulated by tZ7G are EDD1, TUB5, NADP1;1, which have been shown to be CK-regulated in previous studies [55,59,60] (Table 2). Proteins regulated by tZ9G included RPL4 and AT5G43830, which have also previously shown CK-regulation [55,57,58] (Table 2). These data indicate that while tZNGs appear to have some distinct effects on the proteome, treatment with tZNGs may affect the proteome in a manner consistent with previously tested CKs (Table 2). Novel targets of the tZNGs include multiple ribosomal proteins for tZ9G (Fig 6C) and several amino acid metabolism proteins for tZ7G (Fig 6B).

### trans-Zeatin-N-glucosides do not appear to be hydrolyzed to trans-Zeatin in cotyledons

**Fig 7:**
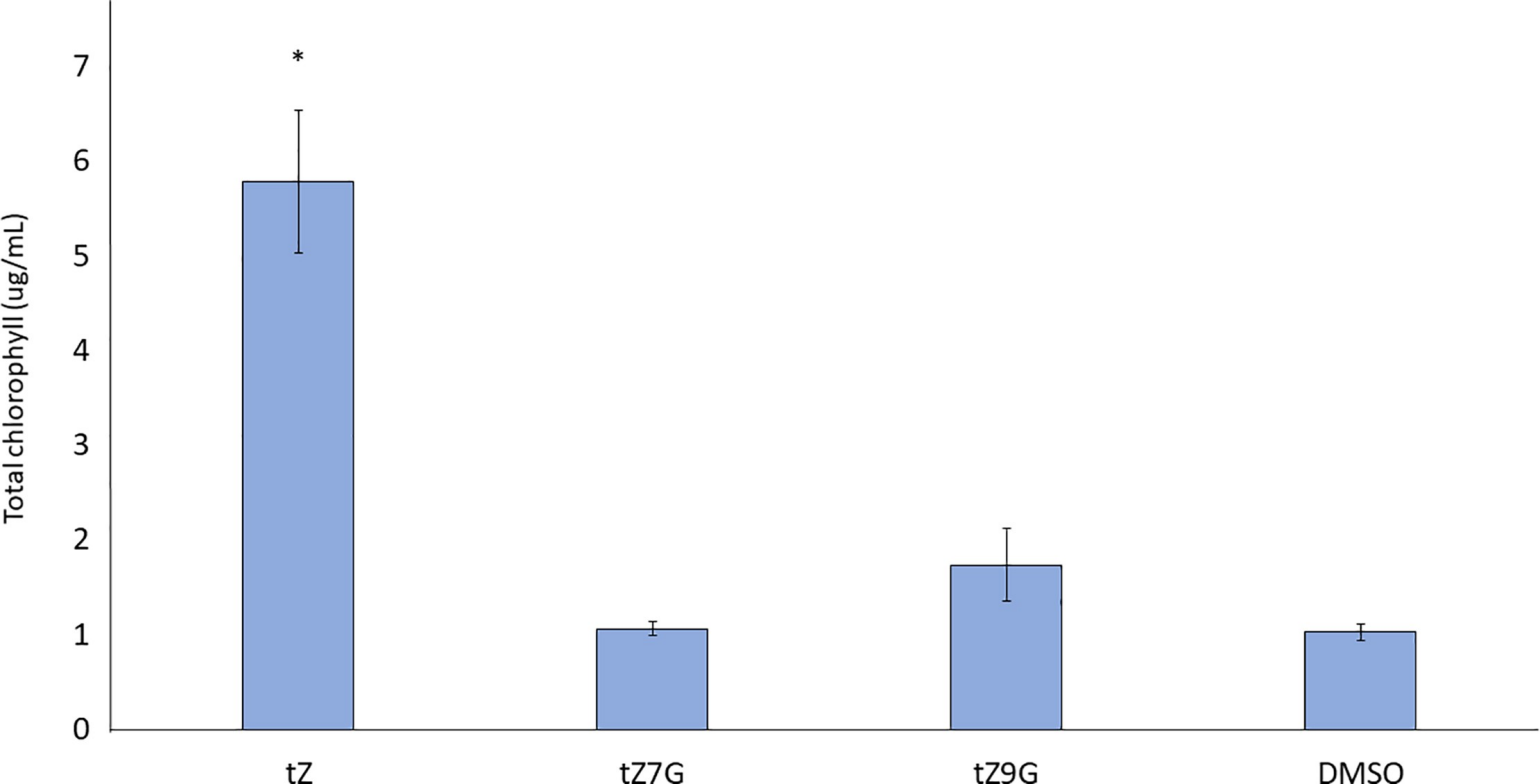
trans-Zeatin-N-glucosides do not delay senescence in trans-Zeatin biosynthetic mutants. Cotyledons from 12dag *cypDM* mutants, which are deficient in tZ-type cytokinins, were subjected to a 6d-long cotyledon senescence assay. While exogenous tZ delayed senescence, tZNGs did not. Average ± SE of four independent biological replicates is presented. *p<0.05, Student’s two-tailed t test.

It was recently reported that within minutes of exogenous application, tZNGs were hydrolyzed to tZ base [27]. While the physiological, transcriptomic, and proteomic data above appear to refute this claim, or at least suggest tZNGs are not fully converted to tZ, we decided to also use a genetic approach to test this hypothesis. Mutants which lack functional copies of CYP735A1 and CYP735A2, the enzymes responsible for hydroxylation of iP-type cytokinins and thus production of tZ-type cytokinins, have been previously generated and described [16]. Use of these mutants in the cotyledon senescence assay, in which tZNGs display the effect most similar to that of tZ, revealed that tZNGs are not capable of delaying senescence in the *cypDM* mutant background, while tZ is (Fig 7). While this result has many implications, it suggests tZNGs are not converted back to tZ in an appreciable amount during this assay. Additionally, a previous study utilized tZ9G obtained from OlChemIm (same manufacturer as the CKs used in this study) to perform a chlorophyll retention assay in wheat; HPLC analysis confirmed no conversion of tZ9G was detected during the assay [31].

## Discussion

### trans-Zeatin-N-glucosides have physiological activity in Arabidopsis

Though often over-generalized as inactivated forms of cytokinin [2,20], findings here indicate that tZNGs appear to have some cytokinin-like capabilities when exogenously applied to Arabidopsis tissues. What is particularly interesting is that the tZNGs tested do not mimic tZ in every assay (Figs 1–4), suggesting that while there is overlap between the activities of these compounds, they are not identical in their biological roles.

One important prerequisite to examining effects of exogenously applied tZNGs is ensuring purity of the compounds. While no synthesized compounds are 100% pure, as noted in the Methods, the tZNGs used in this study were purchased as analytical standards from OlChemIm (Olomouc, Czech Republic) and are >95% pure with no detectable tZ. Previous analysis of these standards found that no tZ was detectable in tZ9G solution before, during, or after conducting chlorophyll retention assays similar to what is described in this study [31]. This, along with the overall results of our study, strongly suggest that any effects observed were due to the exogenous application of untainted tZNGs.

Results from the senescence assay suggest exogenous application of tZ7G and tZ9G is capable of delaying senescence to a similar degree as tZ (Fig 1), and the dose curve similarly reveals it is not only at a micromolar concentration that these compounds display activity (Fig 2). The similar trend of the dose curve for tZ, tZ7G, and tZ9G may suggest the same anti-senescent pathway is being activated in each treatment, though separate mechanisms cannot be ruled out. When the same bioassay was performed using mutants which lack endogenous tZ, tZ7G and tZ9G were not capable of delaying senescence, suggesting activity of exogenously applied tZNGs relies on the presence of endogenous tZ (Fig 7).

In the root growth assay, tZ7G and tZ9G displayed effects different from that of tZ. While tZ significantly inhibited root growth in seedlings, neither tZ7G nor tZ9G altered root length (Fig 3). The difference in effects of tZ and the tZNGs suggests that conversion back to tZ, which has previously been shown to not occur [6,22], is also not a reason for the active effects seen in the senescence assay. While tZ7G and tZ9G mimic tZ during cotyledon senescence, it is possible that these compounds have no effect, or a different effect, on roots. Life stage of the plants may also play a role; the pathway in which tZ7G and tZ9G are perceived may not be active early on during seedling development.

During shoot initiation, tZ7G displayed only minor ability to increase callus mass as opposed to the robust increase in mass seen with tZ (Fig 4). Regeneration did not occur in the tZ9G treatment, nor with the negative auxin-only control. Similar to the root growth assay, these data suggest tZ, tZ7G, and tZ9G do not function identically when exogenously applied to tissue; it is possible each compound only exerts notable effects under particular conditions, i.e. during a specific developmental stage or in a particular organ or tissue.

These bioassay results are strong evidence that tZNGs are not simply converted to tZ as recently reported [27]; one would expect nearly identical results in all bioassays if this were the case. Instead, these data suggest some type of context-dependent cytokinin activity for tZNGs, though the exact nature of this is not yet understood.

### trans-Zeatin-N-glucosides have differing effects on gene expression in Arabidopsis

In the transcriptomic analysis (Fig 5), tZ treatment resulted in gene expression changes quite similar to those seen in other CK transcriptome studies, as many genes from the “Golden list” were affected, and cytokinin-related GO terms were strongly enriched [61,62]. The lack of overlap between tZ with tZ7G and tZ9G, as well as the lack of overlap between the two tZNGs, suggests distinct transcriptional roles for all three compounds. There are some similar targets, however, such as CYTOKININ OXIDASE 4 (CKX4, an extracellular CK degrading enzyme) which is induced by both tZ and tZ7G (Table 1). Though it may seem that induction of CKX4 by tZ7G is possible evidence that CKX4 is capable of degrading CKNGs, previous *in vitro* studies suggest CKNGs are largely resistant to degradation by the AtCKX enzymes, with tZ9G only being degraded by two of seven tested CKXs under acidic conditions and at much lower rates than tZ and tZR [63]. A study in which radiolabeled tZNGs were exogenously applied to soybean tissue revealed that tZNGs do not appear to be degraded *in planta* [22]. Also, ARABIDOPSIS HISTIDINE KINASE 4 (AHK4, CK receptor) is regulated by both tZ and tZ9G, although the two induce different transcript isoforms which differ in their 5’UTR, but not in their protein coding region (Table 1). These data suggest some overlap between the tZ and tZNG perception and/or signaling pathways, despite the largely different transcriptional effects.

One characteristic of tZNGs that should be noted is their size. As mentioned earlier, tZNGs are composed of tZ and a glucose molecule. Therefore, tZNGs are significantly larger than tZ which is likely to impact their transportability. While few CK transporters are known and have not been studied with regards to CKNG transport [15,19,64–66], it may stand to reason that tZNGs are not able to cross membranes into cells as efficiently as tZ. This may be one reason for the different transcriptional effects observed.

An interesting difference between treatments is a difference in subcellular localization of the DEGs. While the subcellular distribution of DEGs between treatments is largely the same, there is a visible increase in percentage of DEGs which localize to the plastid during tZ7G (15% of DEGs) and tZ9G (12.3%) treatment relative to tZ treatment (8.6%) (S1 Fig); this is reflected in GO Enrichment Analysis, as the term “plastid” was significantly enriched in tZ7G DEGs (FDR < 0.014) but was not significant in tZ or tZ9G DEGs. Treatment with tZ7G also has decreased extracellular-localized DEGs (3.7%) relative to treatment with tZ9G (7%) (S1 Fig).

Little overlap was seen between results of the transcriptome and proteome, though this may be unsurprising due to the short treatment time (2h), during which the compounds had to be taken up by the plants, activate signaling pathways, and activate or repress transcription before translation of induced transcripts could take place. The two hour timepoint was chosen as it has previously shown to be effective for determining CK response at the transcriptional level [61,62]. Transcriptional control is of course only one of many levels of gene expression regulation. As noted in other studies of both plant transcriptomes and proteomes, correlation between mRNA and protein levels is influenced by a large number of factors including biological reasons, such as protein turnover, as well as technical reasons, such as difficulty extracting low molecular weight and hydrophobic proteins [67,68]. Future studies will pair early transcriptomes of tZNG-treated plants with proteomes from later timepoints, possibly increasing the overlap between the two datasets, as two hours may not be enough time for the transcriptional changes observed to be observed at the protein level as well. What is striking between the transcriptome and proteome results is how neither show fully unique, nor entirely distinct, effects of the tZNGs; both sets of results show some degree of overlap as well as some unique targets (Fig 6). As noted with the subcellular localization in the transcriptome findings, there are also some differences in subcellular localization of differentially affected proteins identified as being CK-regulated; the proportion of CK-affected proteins which localize to the mitochondria is decreased relative to the total number of proteins identified in the analysis, possibly suggesting organelle-specific responses to CK treatment (S2 Fig).

### Many possible mechanisms may be responsible for CKNG activity

Though CKNGs have not been shown to bind to any tested CK receptors, there may be a number of reasons for this: not all CKNGs have been tested, not all CK receptors have been tested, and the binding experiments have been carried out in microbes which may not be an ideal system for detecting these interactions [5,69,70]. These receptor binding studies have been invaluable to our understanding of CK signaling, but it is noteworthy that BA, a highly potent CK, has shown low binding potential in these assays [69,70] while showing high activity *in planta*. When tested, tZ7G and tZ9G showed no AHK3 or AHK4 binding but still displayed minimal activity *in planta* [70], perhaps pointing toward a role for AHK2 in tZNG perception. This suggests at least the possibility that CKNGs may be acting through the established CK signaling pathway despite no receptor binding assay detecting interaction. However, the lack of similar transcriptional effects between tZ, tZ7G, and tZ9G (Fig 5) may suggest tZNGs, at least at the developmental stages tested, act through different signaling pathways.

It may be possible that the mechanism of CKNG action is a yet undiscovered signaling pathway. Perhaps tZ7G and tZ9G have their own receptors separate from the canonical CK receptors (AHK2, AHK3, and AHK4), though admittedly there is no evidence supporting this hypothesis. However, other histidine kinases such as CYTOKININ INDEPENDENT 1 are known to activate the cytokinin two component signaling pathway despite not being shown to bind to the cytokinins tested [5,71,72], further suggesting our current understanding of the CK signaling pathway is incomplete.

Perhaps the most likely mechanism for the activity of CKNGs is that they act to regulate cytokinin levels. The key cytokinin-N-glucosylating enzyme UGT76C2 has been previously reported to play a role in cytokinin homeostasis [26], and it may stand to reason that exogenous application of CKNGs makes it unfavorable for UGT76C2 to convert CK bases to CKNGs, causing a buildup of CK bases which are responsible for the CK-like phenotypes observed in the senescence assays. One study utilizing an inducible cytokinin oxidase whose preferred substrate is tZ demonstrated that 6h after induction of the enzyme, tZ levels significantly decreased; however, levels of tZ7G surprisingly increased [56]. While it is not clear how increasing degradation of tZ leads to an increase of tZ7G, these data reflect the complex relationship between CK bases and CKNGs and may suggest some type of regulatory role for CKNGs. The authors hypothesize that induction of cytokinin oxidase may have led to an increase in CK biosynthesis; newly synthesized tZ would either be quickly degraded by cytokinin oxidase or converted to the degradation-resistant tZ7G form [56].This may also raise the question of whether CKNGs play some role in stimulating CK biosynthesis, which could be one explanation for the tZ-like effects seen in chlorophyll retention assays (Figs 1 and 2). One approach that could clarify the mechanism by which tZNGs exert effects would be through pairing the CK bioassays with LC-MS measurements to determine CK levels after treatment.

While it is unclear how tZNG might activate an anti-senescent pathway and lead to the delay of senescence in detached cotyledons, it should be noted that DEGs connected to senescence, chloroplast development, and oxidative stress were found to be regulated in the transcriptome analysis (S2 Table). These include some DEGs that overlap with tZ treatment like SEN2/AtCAT3 as well as unique tZNG targets such as PAC and SOD1.

Senescence assays involving the tZ-deficient *cypDM* mutants revealed that tZNGs are not capable of delaying senescence in cotyledons which do not produce endogenous tZ (Fig 7). This suggests that exogenous application of tZNGs alone is not sufficient to delay senescence. This may allude to the possibility that exogenous tZNG application increases endogenous levels of tZ. This possible tZ increase does not appear to be the result of hydrolysis of tZNGs to tZ base, as this would likely result in an effect similar to that of exogenous tZ application, which is not observed (Fig 7). This result also appears to provide genetic evidence countering that described in a recent metabolic study showing conversion of tZNGs to tZ [27]. It must be noted, however, that this assay only included cotyledon tissue; while it does not appear that tZNGs are hydrolyzed to tZ in cotyledons, this data cannot be generalized other organs.

It is important to note that the data presented here do not suggest that the addition of tZNGs always leads to an increase in tZ, as one would expect nearly identical results between all assays if this were the case; tZNG treatment appears to affect plants quite differently from tZ treatment in most experiments (Figs 3–6).

### Further study of CKNG function is necessary

Whatever the mechanism, it is apparent that CKNGs merit further investigation and should not always be assumed to be inactive compounds. Past and current work in cytokinin measurement and phylogenetics has revealed CKNGs are absent or present at extremely low levels in algae and cyanobacteria [73], present at low or moderate levels in some Byrophytes [74], and present at high levels in vascular plants, suggesting an important role for these compounds in the evolution of higher plants [75]. There is almost certainly an evolutionary advantage to explain why plants expend a valuable resource like glucose in making CKNGs, but further investigation is warranted into what exactly these advantages are.

## Supporting information

S1 Table List of all proteins differentially affected by tZ, tZ7G, and tZ9G treatments. Ratio is the relative abundance of protein in the treated sample compared to the DMSO negative control.
(PDF)

S2 Table trans-Zeatin-N-Glucosides regulate the transcriptome uniquely from trans-Zeatin. All transcripts listed here are significantly (padj < 0.05) regulated by tZ7G (yellow) or tZ9G (red).
(TIF)

S3 Table Primer sequences used in qRT-PCR reactions. For analysis, all genes were normalized to PDF1.
(TIF)

S1 Fig Subcellular distribution of DEGs is similar between tZ, tZ7G, and tZ9G treatment. Results from the transcriptome analysis were analyzed for subcellular distribution using SUBA4. Data presented is the percent of DEGs from each treatment group which localize to the noted organelle.
(TIF)

S2 Fig Cytokinin treatment results in a change in localization of detectable proteins. Results from the proteome analysis were analyzed for subcellular distribution using SUBA4. Data in blue represents the percent of differentially affected proteins showing CK responsiveness (i.e. responded to tZ, tZ7G, or tZ9G) which localize to the noted organelle. Data in black indicates the percent of all proteins identified in the analysis (i.e. both CK-responsive and non-CK-responsive) which localize to the noted organelle.
(TIF)

S1 Data Raw data from all presented figures are given in accordance to PLOS data policy. (XLSX)
