# Supplementary material for: trans-Zeatin-N-glucosides have biological activity in Arabidopsis thaliana
Source: PLoS One. 2020 May 7;15(5):e0232762. doi: 10.1371/journal.pone.0232762 (PMC7205299; doi:10.1371/journal.pone.0232762)
Supplement: S1 Table — Ratio is the relative abundance of protein in the treated sample compared to the DMSO negative control. (PDF) [file pone.0232762.s001.pdf]

		tZ		tZ7G		tZ9G	
Accession	Protein name (UniProt)	Ratio	Adj. P-Value	Ratio	Adj. P-Value	Ratio	Adj. P-Value
AT2G45180	rRNA adenine N(6)-methyltransferase	2.9	2.9E-03	1.6	3.1E-01	0.8	7.0E-01
AT2G24940	Probable steroid-binding protein 3	0.2	2.3E-05	0.6	4.3E-01	0.7	9.6E-01
AT3G15450	*Aluminum induced protein with YGL and LRDR motifs*	0.1	3.1E-05	0.5	3.2E-01	0.3	2.2E-01
AT3G15010	UBP1-associated protein 2C	1.6	5.4E-01	3.5	4.5E-02	1.3	5.6E-01
AT1G70580	Glutamate--glyoxylate aminotransferase 2	1.6	1.3E-01	2.9	9.1E-03	1.1	5.3E-01
AT4G38460	Heterodimeric geranylgeranyl pyrophosphate synthase small subunit	1.5	2.0E-01	3.0	4.1E-03	1.0	6.2E-01
AT1G65960	Glutamate decarboxylase 2	1.5	1.3E-01	2.1	4.1E-02	1.0	6.2E-01
AT3G23600	*Alpha/beta-Hydrolases superfamily protein*	1.4	3.4E-01	3.7	4.2E-05	1.1	4.1E-01
AT4G15530	Pyruvate, phosphate dikinase 1	1.4	6.7E-01	4.1	1.4E-02	0.9	8.9E-01
AT2G29450	Glutathione S-transferase U5	1.3	3.0E-01	1.7	3.2E-02	0.9	8.8E-01
AT1G79870	Glyoxylate/hydroxypyruvate reductase A HPR2	1.3	4.3E-01	2.7	1.4E-02	0.7	9.7E-01
AT2G17340	Damage-control phosphatase	1.3	3.6E-02	2.1	8.3E-03	0.6	8.9E-01
AT5G01410	Pyridoxal 5'-phosphate synthase subunit PDX1.3	1.1	6.6E-01	1.8	1.7E-02	0.9	8.6E-01
AT1G17050	Solanesyl diphosphate synthase 2	1.0	8.3E-01	2.4	2.6E-02	1.2	3.5E-01
AT5G65430	14-3-3-like protein GF14 kappa	1.0	8.7E-01	2.8	4.5E-02	1.1	5.8E-01
AT1G23820	Spermidine synthase 1	1.0	7.2E-01	2.2	1.8E-02	1.0	5.3E-01
AT3G48110	Glycine--tRNA ligase	2.8	1.8E-01	4.5	3.8E-02	0.7	9.8E-01
AT4G26110	Nucleosome assembly protein 1	1.1	4.2E-01	0.5	2.7E-02	0.8	8.7E-01
AT1G20010	Tubulin beta-5 chain	0.7	9.8E-01	0.3	3.0E-02	0.7	9.6E-01
AT5G37720	THO complex subunit 4D	0.8	9.7E-01	0.5	2.8E-02	0.7	9.6E-01
AT1G66240	Copper transport protein ATX1	0.7	8.2E-01	0.5	1.3E-02	0.8	9.8E-01
AT1G61570	Mitochondrial import inner membrane translocase subunit TIM13	0.6	9.2E-01	0.3	4.3E-02	0.7	9.6E-01
AT2G38540	Non-specific lipid-transfer protein 1	0.6	5.6E-01	0.4	2.6E-04	0.5	4.6E-01
AT5G48220	*Aldolase-type TIM barrel family protein*	1.2	7.8E-01	1.4	7.8E-01	2.1	2.5E-02
AT4G25100	Superoxide dismutase [Fe] 1	0.8	9.8E-01	0.7	4.3E-01	1.6	1.3E-02
AT4G23890	NAD(P)H-quinone oxidoreductase subunit S	0.7	9.8E-01	1.0	9.9E-01	1.6	1.4E-02
AT1G07320	50S ribosomal protein L4	0.8	9.7E-01	0.7	5.2E-01	0.4	2.4E-02
AT2G35040	*Putative phosphoribosylaminoimidazolecarboxamide formyltransferase*	0.7	9.6E-01	1.0	9.2E-01	0.4	4.6E-02
AT2G04030	Heat shock protein 90-5	0.7	7.8E-01	0.7	5.4E-01	0.4	3.3E-02
AT4G28440	Uncharacterized protein At4g28440	0.7	2.0E-01	0.7	2.4E-01	0.4	5.9E-03
AT1G41880	60S ribosomal protein L35a-2	0.6	8.1E-01	0.8	8.2E-01	0.4	1.7E-02
AT5G27770	60S ribosomal protein L22-3	0.5	1.3E-01	0.6	1.5E-01	0.4	1.4E-02
AT4G29060	*Elongation factor Ts*	0.5	3.8E-01	0.7	3.8E-01	0.3	6.7E-05
AT4G18370	Protease Do-like 5	0.3	2.2E-01	0.4	2.1E-01	0.2	1.1E-02
AT5G43830	*Aluminum induced protein with YGL and LRDR motifs*	0.9	9.1E-01	1.1	8.8E-01	0.4	3.8E-02
AT1G56110	*Nop domain-containing protein*	4.2	3.8E-03	2.3	2.0E-01	1.0	8.1E-01
AT2G31810	Acetolactate synthase small subunit 2	4.0	2.3E-02	3.1	1.8E-01	1.4	5.6E-01
AT4G14910	Imidazoleglycerol-phosphate dehydratase 2	2.9	5.5E-02	4.1	3.5E-02	1.1	7.0E-01
AT5G62530	Delta-1-pyrroline-5-carboxylate dehydrogenase 12A1	2.8	2.8E-02	3.1	2.6E-02	0.9	9.1E-01
AT5G66090	*Cell wall integrity/stress response component*	2.7	5.7E-01	6.4	2.9E-04	2.3	1.4E-01
AT1G05385	Photosystem II D1 precursor processing protein PSB27-H2	2.7	2.2E-05	3.3	1.4E-08	1.4	3.4E-02
AT3G27360	Histone H3.2	2.3	3.4E-02	9.3	2.5E-16	1.0	6.8E-01
AT4G36810	Heterodimeric geranylgeranyl pyrophosphate synthase large subunit 1	2.1	3.8E-03	2.2	4.6E-02	1.1	3.7E-01
AT5G11670	NADP-dependent malic enzyme 2	2.1	1.3E-03	1.8	4.5E-02	1.1	5.2E-01
AT5G10920	Argininosuccinate lyase	2.0	1.4E-02	2.3	3.2E-02	1.1	3.6E-01
AT2G10940	*Bifunctional inhibitor/lipid-transfer protein/seed storage 2S albumin superfamily protein*	2.0	1.9E-01	2.3	1.7E-05	1.4	1.1E-01
AT2G30200	*EMBRYO DEFECTIVE 3147*	1.7	2.6E-02	2.3	3.2E-03	1.3	8.5E-02
AT2G26340	*Uncharacterized protein*	1.7	5.7E-02	2.9	9.9E-03	1.1	5.5E-01
AT1G04270	40S ribosomal protein S15-1	1.7	4.4E-02	1.7	5.5E-02	1.0	6.5E-01
AT1G04690	Probable voltage-gated potassium channel subunit beta	1.6	5.5E-02	3.0	3.7E-04	1.0	5.6E-01
AT3G08940	Chlorophyll a-b binding protein CP29.2	1.8	1.9E-02	2.8	1.1E-06	1.0	6.8E-01
AT3G22960	Plastidial pyruvate kinase 1	1.9	5.6E-03	1.8	6.6E-02	1.0	6.3E-01
AT5G13450	ATP synthase subunit O	1.5	1.2E-01	2.1	2.0E-03	1.1	5.0E-01
AT1G13930	*Oleosin-B3-like protein*	0.5	1.4E-01	0.5	8.2E-03	0.6	6.7E-01
AT2G47710	*Adenine nucleotide alpha hydrolases-like superfamily protein*	0.6	5.8E-01	0.5	2.0E-02	0.7	9.4E-01
AT3G52500	*Eukaryotic aspartyl protease family protein*	0.3	2.7E-03	0.6	2.4E-01	0.7	9.5E-01
AT3G02780	Isopentenyl-diphosphate Delta-isomerase II	0.2	3.3E-02	0.5	4.6E-01	0.7	9.7E-01
AT2G38140	30S ribosomal protein S31	0.4	1.3E-01	0.7	5.4E-01	0.4	1.6E-02
AT2G41530	S-formylglutathione hydrolase	0.6	6.3E-01	0.8	8.8E-01	0.3	3.5E-03
AT3G07720	*Galactose oxidase/kelch repeat superfamily protein*	0.5	4.4E-01	0.7	5.6E-01	0.4	3.0E-02
AT3G09500	60S ribosomal protein L35-1	0.4	1.4E-02	0.7	3.7E-01	0.5	4.7E-01
AT3G48930	40S ribosomal protein S11-1	0.4	5.5E-02	0.7	4.4E-01	0.5	2.4E-01
AT2G38040	Acetyl-coenzyme A carboxylase carboxyl transferase subunit alpha	0.4	1.8E-01	1.0	9.8E-01	0.3	1.5E-02
AT2G40740	WRKY transcription factor 55	0.2	2.5E-04	0.8	9.2E-01	0.4	1.0E-01
AT4G29590	*S-adenosyl-L-methionine-dependent methyltransferases superfamily protein*	0.2	2.6E-04	0.8	9.0E-01	0.2	3.1E-04
AT5G66680	Dolichyl-diphosphooligosaccharide--protein glycosyltransferase 48 kDa subunit	2.9	1.9E-03	2.8	3.0E-02	1.7	2.3E-01
AT5G47030	ATP synthase subunit delta	2.4	1.0E-04	4.1	2.0E-07	1.8	3.5E-04
AT4G10450	60S ribosomal protein L9-2	2.1	1.6E-01	1.8	9.4E-01	2.1	7.9E-03
