# Supplementary figures and images for: trans-Zeatin-N-glucosides have biological activity in Arabidopsis thaliana
Source: PLoS One. 2020 May 7;15(5):e0232762. doi: 10.1371/journal.pone.0232762 (PMC7205299; doi:10.1371/journal.pone.0232762)

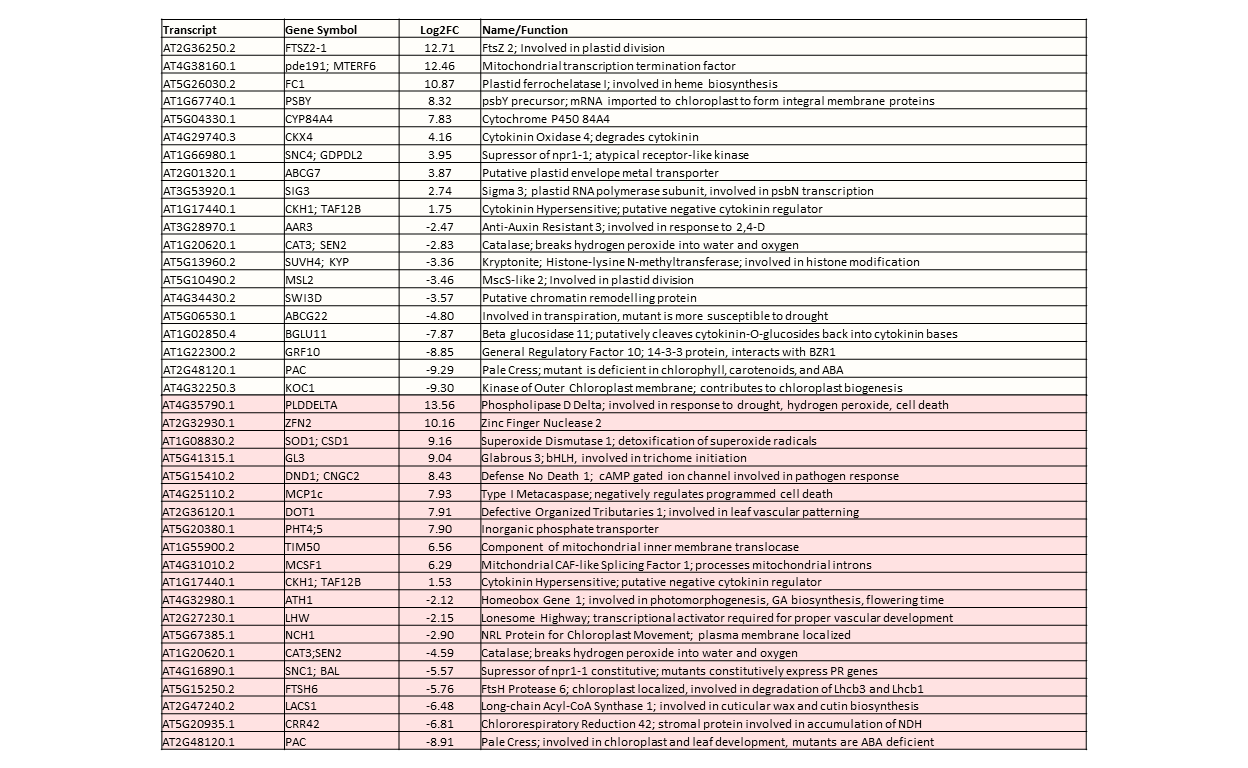

Supplement: S2 Table — All transcripts listed here are significantly (padj < 0.05) regulated by tZ7G (yellow) or tZ9G (red). (TIF) [file pone.0232762.s002.tif]

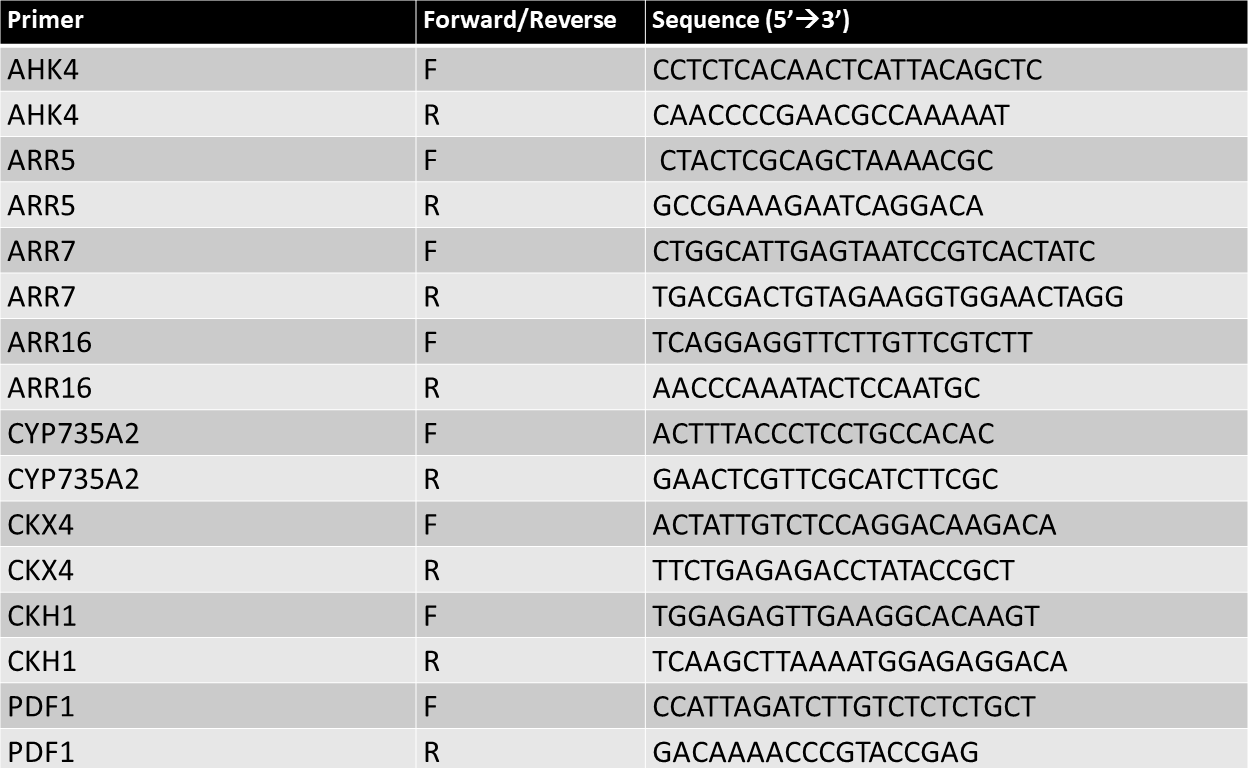

Supplement: S3 Table — For analysis, all genes were normalized to PDF1. (TIF) [file pone.0232762.s003.tif]

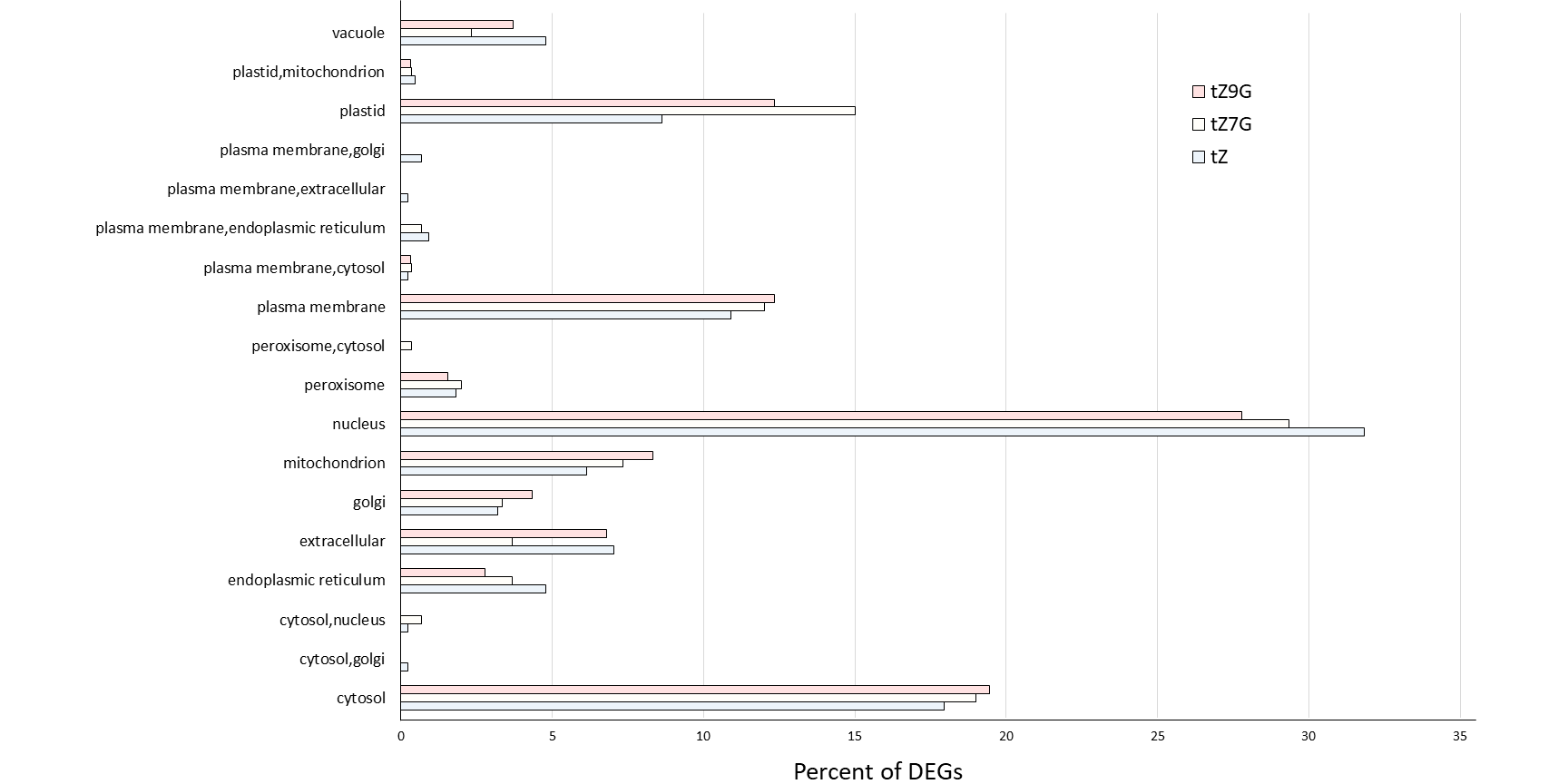

Supplement: S1 Fig — Results from the transcriptome analysis were analyzed for subcellular distribution using SUBA4. Data presented is the percent of DEGs from each treatment group which localize to the noted organelle. (TIF) [file pone.0232762.s004.tif]

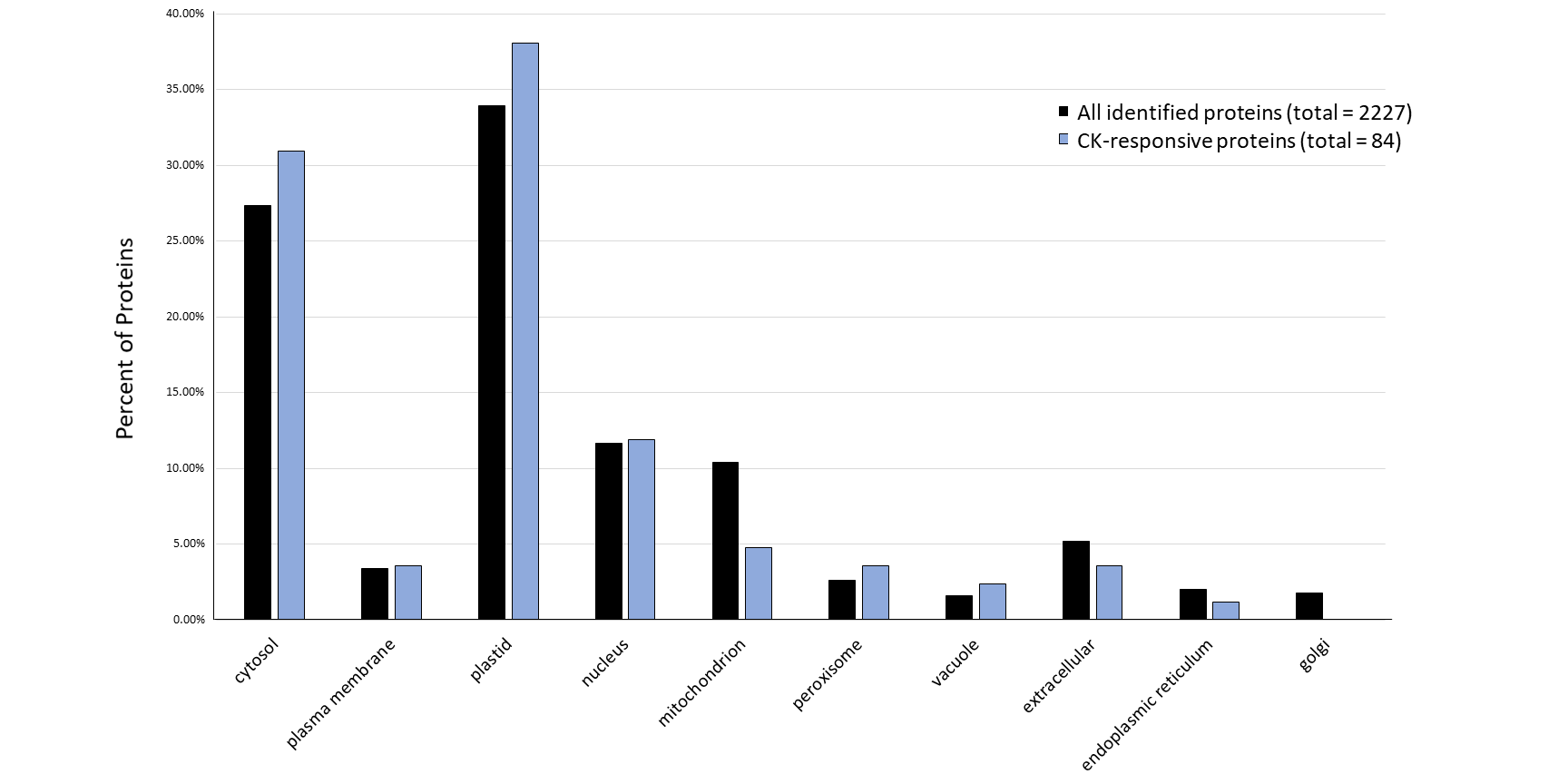

Supplement: S2 Fig — Results from the proteome analysis were analyzed for subcellular distribution using SUBA4. Data in blue represents the percent of differentially affected proteins showing CK responsiveness (i.e. responded to tZ, tZ7G, or tZ9G) which localize to the noted organelle. Data in black indicates the percent of all proteins identified in the analysis (i.e. both CK-responsive and non-CK-responsive) which localize to the noted organelle. (TIF) [file pone.0232762.s005.tif]
